# Perceptions and Practices of Pediatric Dentists on Clear Aligner Therapy in Mixed Dentition: A Cross-Sectional Survey

**DOI:** 10.7759/cureus.89258

**Published:** 2025-08-02

**Authors:** Amirdha Shahithya, Kavitha Swaminathan, Jagadheeswari Ramamoorthy, KC Vignesh, Vivek K, Selvakumar Haridoss

**Affiliations:** 1 Dentistry, Sri Ramachandra Institute of Higher Education and Research, Chennai, IND; 2 Pediatric and Preventive Dentistry, Sri Ramachandra Institute of Higher Education and Research, Chennai, IND

**Keywords:** aesthetic dentistry, aligner therapy, clear aligners, clinical perception, interceptive orthodontics, mixed dentition, orthodontic treatment, pediatric dentistry, survey-based study, tooth movement

## Abstract

Introduction

Clear aligners have gained traction in pediatric orthodontics due to their aesthetic and hygienic advantages. However, their applicability and effectiveness in mixed dentition remain underexplored. This study aims to assess the pediatric dentists' perceptions, practices, and challenges associated with clear aligner therapy in mixed dentition.

Methods

A cross-sectional survey was conducted among 300 pediatric dentists in India using a validated 17-item online questionnaire. The questionnaire assessed demographic data, clinical experiences, perceived benefits, challenges, and future intent regarding clear aligner use. Data were analyzed using descriptive statistics and chi-square tests to evaluate associations between clinical experience and aligner-related practices.

Results

Among the 300 respondents, 63.9% had never used clear aligners in mixed dentition, and 61.1% expressed probable future use. Better aesthetics (69.4%) and improved hygiene (55.6%) were the most cited benefits. Major challenges included high cost (19.4%) and difficulty in managing erupting teeth (13.9%). No significant association was found between the dentist's years of experience and previous (p=0.0917) or future use of aligners (p=0.434).

Conclusion

Pediatric dentists perceive clear aligners as a moderately effective tool during mixed dentition, with parental demand and aesthetic benefits driving interest. However, limitations related to cost, compliance, and control of tooth movement warrant further longitudinal and outcome-based studies before routine adoption.

## Introduction

Clear aligner therapy has significantly evolved over the past two decades, and was initially designed to manage mild-to-moderate orthodontic issues in adult populations [1]. The introduction of advanced software algorithms and improved materials has enabled their expanded use in more complex orthodontic cases, including pediatric applications during mixed dentition phases. Specifically, recent innovations such as eruption compensation wells and precision attachments have facilitated the application of clear aligners in younger patients [1]. This development has addressed earlier limitations, allowing aligner treatment to accommodate dynamic changes during tooth eruption and exfoliation effectively.

Several studies have evaluated the benefits and limitations of clear aligners compared to traditional fixed appliances in pediatric orthodontics. Clear aligners have demonstrated superior oral hygiene, less gingival inflammation, and better periodontal health outcomes compared to fixed orthodontic appliances, primarily due to ease of oral hygiene maintenance [2]. Moreover, clear aligner therapy has been associated with less discomfort, fewer dietary restrictions, reduced emergency visits, and increased patient acceptance owing to its aesthetics and convenience [3,4]. However, despite these advantages, evidence regarding the predictability of certain tooth movements and overall effectiveness in interceptive orthodontic treatment remains limited, highlighting a crucial gap in pediatric orthodontic literature [5,6].

Given the rising popularity of clear aligners among pediatric dentists, there is limited high-quality evidence evaluating their effectiveness and practitioners' perceptions during mixed dentition. This study investigates the perceptions, clinical experiences, and challenges faced by pediatric dentists in India regarding the use of clear aligner therapy during the mixed dentition period. This investigation will contribute critical insights to guide clinical decision-making, development of aligner technology, and future orthodontic practice standards in pediatric dentistry.

## Materials and methods

Study design and ethical approval

This was a descriptive, questionnaire-based, cross-sectional study conducted among pediatric dentists across India. The survey was conducted across India, targeting pediatric dentists from diverse practice settings, including academic institutions, private clinics, and government hospitals, to capture a nationally representative perspective. Ethical approval for the study was obtained from the Institutional Review Board of Sri Ramachandra Institute of Higher Education and Research (CSP-III/25/APR/20/221). The study adhered to the ethical principles outlined in the Declaration of Helsinki.

Study duration and participants

The survey was conducted over a period of three months from April 2025 to June 2025. Practicing pediatric dentists from various clinical and academic settings were invited to participate. Inclusion criteria comprised currently practicing pediatric dentists who consented to participate in the study. Retired dentists, general practitioners, and other specialists were excluded.

Sample size determination

The sample size was calculated using nMaster software (version 2, Christian Medical College (CMC) Vellore, India) based on a 95% confidence level (α = 0.05), 80% power (β = 0.20), and a precision of 6%, assuming a 50% response distribution. The minimum required sample size was estimated to be 267 [7]. To ensure representation and allow for subgroup analyses, the questionnaire was distributed until 300 complete responses were obtained.

Survey instrument

The 17-item questionnaire (given in the Appendix) was designed following a comprehensive literature review and covered four domains: demographics, clinical practice, perceptions, and challenges in using clear aligners in mixed dentition. Face and content validity were established by a panel of five pediatric dentistry experts who evaluated the items for relevance and clarity.

A pilot test was conducted with 15 pediatric dentists (excluded from final analysis) to assess usability, layout, and average completion time. Based on their input, minor refinements were made. The finalized questionnaire demonstrated good internal consistency with a Cronbach’s alpha score of 0.82 for multi-item sections.

The questionnaire included both closed-ended and open-ended questions and was pretested for clarity and relevance. A structured questionnaire comprising 17 items across four domains - demographics, perceptions, challenges, and satisfaction with clear aligner therapy - was developed and validated through expert review. 

Data collection procedure

The questionnaire was circulated via Google Forms through society mailing lists, institutional alumni WhatsApp groups, social media platforms, and academic dental networks across India. The survey remained open for six weeks, during which time weekly reminders were sent. Pediatric dentists were encouraged to share the form with colleagues (snowball sampling). High engagement through professional peer networks, direct institutional endorsements, and mobile-friendly formats facilitated an exceptionally high response rate. Similar studies in India have documented response rates of 85-95% using digital channels and targeted outreach [8,9]. Participation was voluntary, and informed consent was obtained at the beginning of the questionnaire. Duplicate responses were prevented by restricting multiple entries from the same email address.

Scoring criteria and data analysis

Most questionnaire items were categorical (nominal or ordinal) and analyzed using frequency and percentage distributions. Responses were not aggregated into a composite score, as the aim was to assess individual trends in perceptions, practices, and challenges. Therefore, scoring criteria were not applied, and each item was treated independently for analysis.

Two open-ended questions were included at the end of the survey to elicit qualitative insights on improvements in aligner therapy and additional suggestions. These responses were analyzed using thematic content analysis. Responses were read independently by two reviewers, and emerging themes were coded and grouped. Any disagreements were resolved through consensus.

Statistical analysis

Data were analyzed using IBM SPSS Statistics for Windows, Version 26 (Released 2019; IBM Corp., Armonk, New York, United States). Descriptive statistics, including frequencies and percentages, were used to summarize categorical variables. Inferential statistics, such as Chi-square tests, were applied to assess associations between selected variables (e.g., clinical experience and perceptions of clear aligner therapy). As the dataset consisted entirely of categorical variables, and no parametric tests were employed, normality testing was not applicable. A p-value of <0.05 was considered statistically significant.

## Results

A total of 300 pediatric dentists participated in the survey. The majority of respondents were aged 25-35 years (n=217, 72.3%), followed by 36-45 years (n=50, 16.7%), and 46-55 years (n=33, 11.1%). Female participants comprised 69.4% (n=208) of the total sample, while male participants accounted for 30.6% (n=92). Regarding years of clinical experience, most participants had less than five years (n=200, 66.7%), followed by five to 10 years (n=58, 19.3%), and 11-15 years (n=42, 14.0%). In terms of practice setting, the majority were affiliated with academic institutions (n=192, 63.9%), followed by private practice (n=83, 27.8%), government institutions (n=17, 5.7%), and other settings (n=8, 2.6%). A summary of the responses across demographic variables and clinical perceptions is presented in Table 1.

**Table 1:** Frequency distribution of participant responses (n=300)

Variable	Category	Frequency (n)	Percentage (%)
Age	25-35 years	217	72.3
	36-45 years	50	16.7
	46-55 years	33	11
Gender	Female	208	69.3
	Male	92	30.7
Years of experience	<5 years	200	66.7
	11-15 years	42	14
Practice setting	Academic	192	64
	Private	83	27.7
Used aligners	No	192	64
	Considering	75	25
	Yes	33	11
Recommendation frequency	Occasionally	108	36
	Never	92	30.7
Reasons for using aligners	Better aesthetics	208	69.3
	Improved hygiene	167	55.7
	Fewer visits	117	39
Effectiveness	Moderately effective	200	66.7
	Very effective	92	30.7
Challenges	High cost	58	19.3
	Erupting teeth	42	14
	Compliance issues	33	11
Treatment duration	Longer than fixed	133	44.3
	About the same	100	33.3
Patient satisfaction	Moderately satisfied	117	39
	Very satisfied	92	30.7
Clinician satisfaction	Moderately satisfied	117	39
	Very satisfied	92	30.7
Future use	Probably	183	61
	Definitely	108	36
	Not at all	9	3

**Figure 1:**
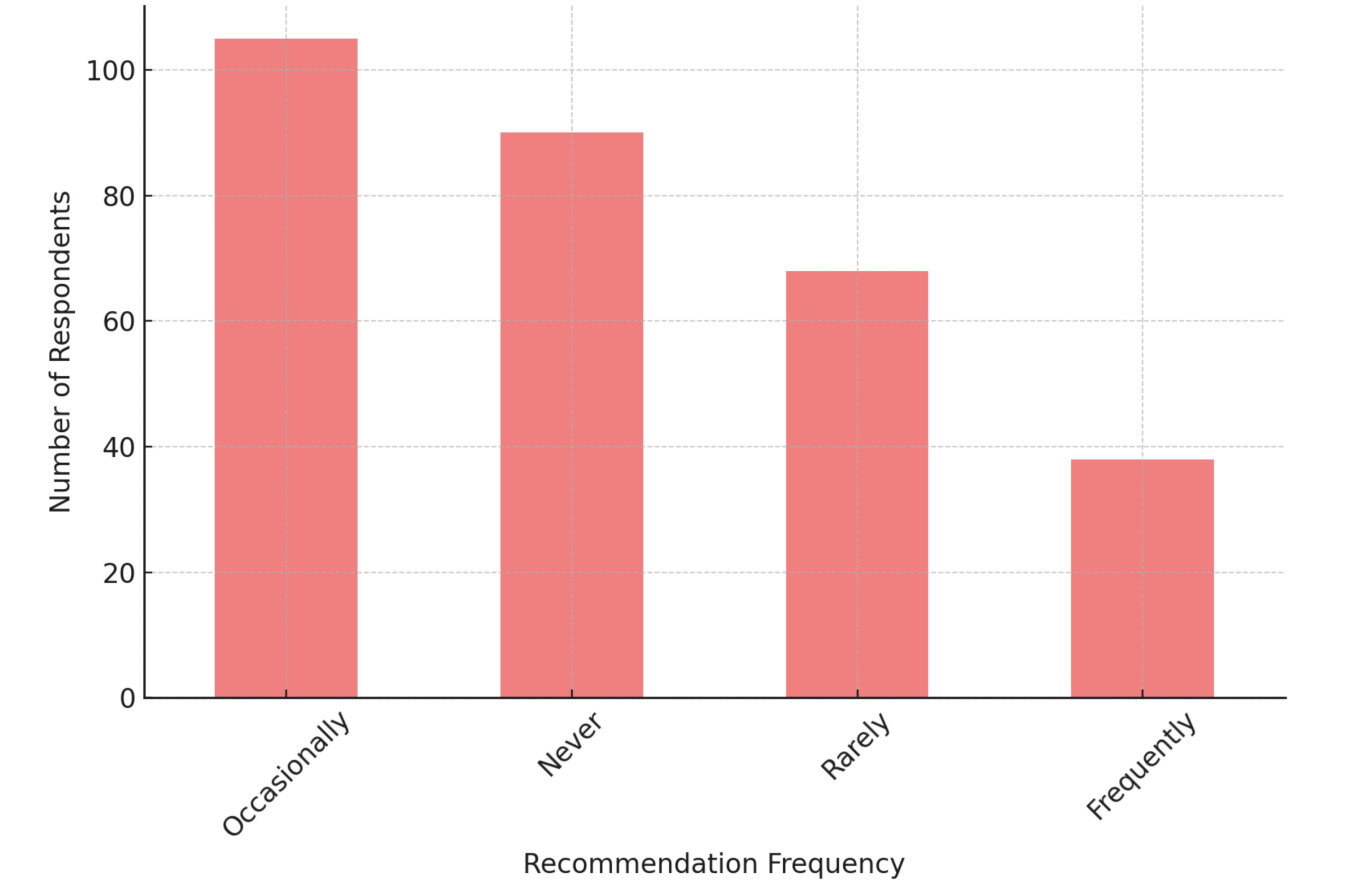
Frequency of recommendation for clear aligner therapy

Concerning the use of clear aligners in mixed dentition cases, 63.9% (n=192) of pediatric dentists reported they had not used clear aligners, whereas 25% (n=75) were considering their use, and only 11.1% (n=33) had used them previously. Regarding how often clear aligners are recommended, 36.1% (n=108) reported recommending them occasionally, followed by 30.6% (n=92) who never recommended them, 22.2% (n=67) who recommended them rarely, and 11.1% (n=33) who recommended them frequently. The distribution of the frequency of recommendation is illustrated in Figure 1.

The most commonly cited reasons for using clear aligners in mixed dentition were better aesthetics (n= 208, 69.4%), improved oral hygiene (n=167, 55.6%), and fewer clinical visits (n=117, 38.9%). Regarding perceived effectiveness, moderate effectiveness was reported by 200 respondents (66.7%), and very effective ratings were given by 92 participants (30.6%). The primary challenges identified included high cost (n=58, 19.4%), difficulty managing erupting teeth (n=42, 13.9%), and compliance issues (n=33, 11.1%). When comparing treatment durations, 133 respondents (44.4%) believed that clear aligner therapy took longer than fixed appliances, while 100 (33.3%) felt the duration was about the same.

**Figure 2:**
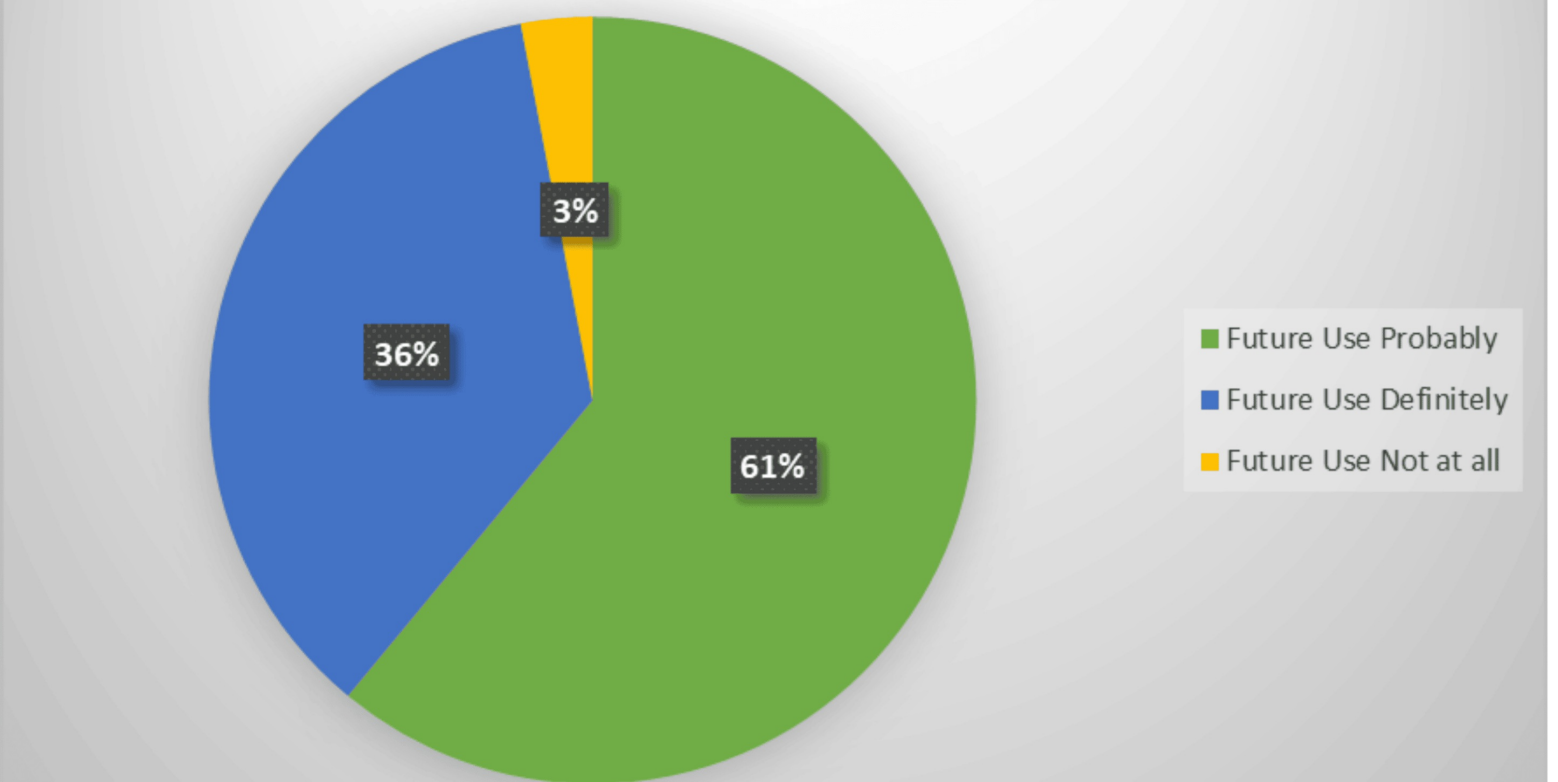
Future intention to use clear aligners

Patient satisfaction with aligner therapy was rated as moderately satisfied by 117 respondents (38.9%) and very satisfied by 92 (30.6%). Clinician satisfaction mirrored this pattern. Regarding future intent, 183 participants (61.0%) stated they would probably continue using aligners in mixed dentition, while 108 (36.0%) expressed definite intent. These response distributions are illustrated in Figure 2.

A chi-square test of association between years of experience and previous use of clear aligners showed no statistically significant association (χ²=10.89, df=6, p=0.0917). Similarly, no significant association was found between the practitioner's years of experience and intention to use aligners in the future (χ²=5.90, df=6, p=0.434), as shown in Table 2.

**Table 2:** Association between years of experience and aligner usage (n=300) χ²: Chi-square statistic; df: degrees of freedom.

Comparison	Chi-square (χ²)	Degrees of freedom (df)	p-value	Interpretation
Experience vs. previous use	10.89	6	0.0917	Not significant, but suggestive
Experience vs. future intent to use	5.9	6	0.434	Not significant, no clear relationship

## Discussion

Clear aligners have become increasingly popular in pediatric orthodontics due to their aesthetic appeal, comfort, and improved oral hygiene maintenance compared to traditional appliances [10]. The development of systems like Invisalign First^TM^ has expanded the use of clear aligners to younger populations in the mixed dentition phase, enabling interceptive management of malocclusions with improved patient acceptance and compliance [11]. The current study reflects this shift, highlighting the increasing interest among Indian pediatric dentists toward clear aligner use in children.

A recent scoping review by Chandra et al. (2024) supports the notion that aligners offer clinical advantages in mixed dentition, particularly regarding improved plaque control, reduced chairside time, and lower postoperative discomfort [12]. These factors may explain the moderate-to-high clinician and patient satisfaction observed in our study. Furthermore, literature shows that aligners improve patient-reported outcomes, including speech comfort and aesthetics, which are critical in pediatric populations [13,14]

In terms of perceived effectiveness, most participants in this study rated aligners as moderately effective, aligning with clinical trials that show mixed results in achieving complex movements like extrusion, rotation, and root torque (Kim et al., 2023; Loberto et al., 2022) [11,15]. As noted by Wang et al. in a clinical consensus, the limited predictability of certain movements, especially during early transitional dentition, remains a significant limitation [16].

However, our findings are consistent with cautionary trends reported in previous clinician surveys. For instance, a national survey of orthodontists revealed limited adoption of clear aligner therapy during the mixed dentition stage, with only 18.1% reporting active use, largely due to concerns surrounding eruption control and the predictability of tooth movements [1]. Similarly, expert consensus has underscored that certain biomechanical movements, particularly extrusion and rotation, continue to pose challenges with the current aligner systems, thereby substantiating the reservations expressed by a proportion of our respondents [17].

Despite increasing awareness and interest, the adoption of aligners appears uneven across practitioner demographics. Our results indicate a more favorable attitude among younger pediatric dentists, particularly those under 35 years, with a significantly higher intent to use aligners in future cases. This aligns with Inchingolo et al. (2024), who found that digital competency and recent exposure to aligner-based curricula were associated with greater clinical openness [18]. In contrast, more experienced practitioners demonstrated conservative usage patterns, possibly due to longstanding reliance on fixed appliances and concerns regarding erupting dentition control.

Interestingly, a study by Dianiskova et al. (2023) noted that while parental interest in aligners for children is high, many clinicians remain hesitant due to concerns over cost, compliance, and managing partially erupted teeth [19]. These concerns mirror those found in our cohort, where cost and compliance were frequently cited as limiting factors. Additionally, our finding that aligner therapy was perceived as having longer treatment times than fixed appliances contrasts with studies showing comparable or shorter durations in selected cases [11,20] .

Limitations

As with all cross-sectional surveys, causality cannot be inferred from the observed associations. The potential for response and selection bias is present due to the self-administered nature of the online questionnaire. Moreover, while this study used a standardized sample of 300 responses derived from an initial validated set, generalizability may be constrained by the lack of regional stratification. Importantly, no objective clinical outcome data were captured, which limits the depth of the clinical interpretation.

Strengths

Despite these limitations, this is one of the first Indian studies to systematically assess pediatric dentists' perspectives on aligner use in mixed dentition. The questionnaire covered critical domains, including perceived effectiveness, satisfaction, and barriers. Methodological rigor was maintained through STrengthening the Reporting of OBservational studies in Epidemiology (STROBE) compliance and expert validation of the instrument [21].

Future directions

There is a need for longitudinal, multicenter clinical studies to evaluate treatment outcomes of clear aligners in early mixed dentition. Incorporating objective metrics such as alignment accuracy, treatment duration, and relapse rates will enhance evidence-based decision-making. To improve the adoption of clear aligners during mixed dentition, professional dental societies should develop clinical guidelines tailored to pediatric populations, incorporate aligner education in postdoctoral curricula, and facilitate cost-effective collaborations with manufacturers.

## Conclusions

Clear aligner therapy in mixed dentition is gaining interest among pediatric dentists, particularly younger practitioners, due to its aesthetic and hygienic advantages. While clinical satisfaction and perceived effectiveness remain moderate, concerns such as high cost, compliance issues, and eruption management persist. No significant associations were found between years of experience and aligner usage, indicating a broader acceptance trend across age groups. To support evidence-based adoption, pediatric dental societies should consider developing training modules, clinical guidelines, and educational workshops that address aligner use in mixed dentition and improve clinician readiness for this evolving modality.

## Appendices

Questionnaire

Demographic Information:

1. Age:

25-35 years

36-45 years

46-55 years

56 years and above

2. Gender:

Male

Female

Prefer not to say

3. Years of experience in Pediatric Dentistry:

Less than 5 years

5-10 years

11-15 years

More than 15 years

4. Practice Setting:

Private Practice

Academic Institution

Government Institution

Other (Please specify):

Perceptions on Clear Aligners in Mixed Dentition:

5. Have you used clear aligners for treating patients in mixed dentition?

Yes

No

Considering it

6. How often do you recommend clear aligner therapy for mixed dentition patients?

Frequently

Occasionally

Rarely

Never

7. What are the primary reasons for using clear aligners in mixed dentition? (Select all that apply)

Better aesthetics

Better oral hygiene

Less discomfort

Fewer visits

Parental demand

Other (Please specify):

8. Do you believe clear aligners are effective in controlling tooth movement in mixed dentition?

Very effective

Moderately effective

Less effective

Ineffective

9. What types of malocclusion are best treated with clear aligners in mixed dentition? (Select all that apply)

Class I malocclusion

Class II malocclusion

Class III malocclusion

Crowding

Spacing

Others (Please specify):

Challenges With Clear Aligners:

10. What are the main challenges you face when using clear aligners in mixed dentition? (Select all that apply)

Inefficient tooth movement

Compliance issues

High cost

Limited indications

Managing erupting teeth

Other (Please specify):

11. Is treatment time with clear aligners longer than with fixed appliances?

Yes

No

About the same

12. Are clear aligners cost-effective for patients in mixed dentition compared to fixed appliances?

Yes

No

Depends on the case

General Opinions:

13. How satisfied are your patients with clear aligner therapy in mixed dentition?

Very satisfied

Moderately satisfied

Neutral

Unsatisfied

14. How satisfied are you with the results of clear aligner therapy in mixed dentition?

Very satisfied

Moderately satisfied

Neutral

Unsatisfied

15. Would you continue to use clear aligners for mixed dentition in the future?

Definitely

Probably

Unlikely

Not at all

16. What improvements would you like to see in clear aligner technology for mixed dentition patients? (Open-ended)

17. Additional comments or suggestions regarding clear aligner therapy in mixed dentition. (Open-ended)
